# Short-chain fatty acids butyrate and acetate limit Zika virus replication and associated ocular manifestations via the G-protein coupled receptor 43/FFAR2

**DOI:** 10.1128/jvi.01826-25

**Published:** 2025-12-08

**Authors:** Nikhil Deshmukh, Prince Kumar, Lal Krishan Kumar, Vaishnavi Balendiran, Pawan Kumar Singh

**Affiliations:** 1 Department of Ophthalmology, Mason Eye Institute, University of Missouri School of Medicine 12271, Columbia, Missouri, USA; The Ohio State University, Columbus, Ohio, USA

**Keywords:** Zika virus, butyrate, acetate, inflammation, eye, trabecular meshwork, intraocular pressure, retinal atrophy, glaucoma

## Abstract

Short-chain fatty acids (SCFAs) are gut microbial metabolites produced by gut microbiota from dietary fiber. SCFAs have shown both pro- and anti-viral roles among different viruses and are known to regulate immune functions during infections. However, their role against the Zika virus (ZIKV), in general, and ocular infection, in particular, has never been investigated. In the present study, we aimed to examine the role of three SCFA derivatives: phenylbutyrate (PBA), sodium butyrate (NaB), and sodium acetate (NaAC), on ZIKV replication and associated ocular complications using primary human trabecular meshwork cells (HTMCs) and an IFNAR1-deficient mouse model of ocular infection. Our findings reveal that PBA and NaAc treatment dramatically suppressed the ZIKV replication in HTMCs. NaB showed a slightly less effect than PBA and NaAc. PBA and NaAc treatment significantly attenuated the ZIKV-induced inflammatory cytokine, interferons, and interferon-stimulated genes response via antagonizing the RIG-I/NFκB/MAPKs/STAT1-3 signaling pathways. We discovered that ZIKV induces the expression of free fatty acid receptor 2 (FFAR2)/GPR43 in HTMCs, which is further potentiated by PBA/NaAc. Pharmacological inhibition of FFAR2 abrogated the protective abilities of PBA/NaAc and significantly increased viral replication. Blocking FFAR2 receptors promoted ZIKV-induced cell death, which was suppressed by PBA and NaAc. Butyrate and acetate also inhibited ZIKV binding and cellular entry and inactivated the virus before internalization. PBA and NaAc treatment in mice attenuated the ZIKV-induced ocular manifestations (intraocular pressure, RPE/retinal atrophy, and TM/anterior segment inflammation), which was abrogated by FFAR2 inhibition by 4-CMTB, a selective pharmacological inhibitor of FFAR2. Collectively, our findings indicate that SCFA treatment is an effective approach to limit ZIKV replication and associated ocular damage and may be worth exploring as a means to treat or prevent ZIKV-induced ocular complications/glaucoma in humans.

**IMPORTANCE:**

ZIKV is known to cause severe ocular manifestations in *in-utero* exposed infants; however, the molecular mechanisms of ZIKV-induced ocular complications remain unknown. SCFAs have demonstrated both pro- and anti-viral roles against different viruses; however, their role against ZIKV is unknown. We showed that SCFAs butyrate and acetate suppress ZIKV transmission and associated ocular complications. The anti-ZIKV activity of these SFACs is mediated via FFAR2, and pharmacological inhibition of FFAR2 promotes ZIKV-induced inflammatory and cell death responses, as well as ocular malformations.

## INTRODUCTION

Zika virus (ZIKV) is a global public health threat due to its association with severe neurological and ocular manifestations collectively referred to as congenital Zika syndrome (1). ZIKV gained global attention in 2015 via an epidemic in the Americas that affected millions of people, resulting in the declaration of a Public Health Emergency of International Concern (PHEIC) by WHO (2–4). Although PHEIC was lifted in November 2016, ZIKV remains a global concern due to its ongoing transmission in some geographical areas and potential long-term effects on affected individuals. Currently, ZIKV is circulating in >89 countries, and sporadic outbreaks are reported in different regions around the world. Therefore, constant surveillance is essential to ensure preparedness for early detection and treatment of future ZIKV pandemics (5, 6). ZIKV is a positive-sense RNA virus that belongs to the *Flaviviridae* family. It is an arbovirus and is primarily transmitted through mosquito bites. However, it can also spread via sexual intercourse, blood transfusion, organ transplantation, and maternal-fetal transmission (7, 8). *In utero* exposure to ZIKV is linked to severe microcephaly, vision and hearing impairment, as well as articular and musculoskeletal abnormalities (1, 9). Among ocular manifestations, ZIKV caused a wide range of ocular symptoms, including chorioretinal atrophy, hypoplasia, focal pigmented mottling, RPE mottling, retinal focal spots, severe retinal vessel attenuation, optic nerve atrophy, optic disc anomalies, and congenital glaucoma (10–16). However, the ocular disease pathogenesis and long-term consequences of ZIKV infection remain unknown. Besides, no specific antiviral therapies or vaccines are currently available for ZIKV; therefore, it is essential to investigate alternative antivirals to treat/prevent ZIKV-induced complications.

Short-chain fatty acids (SCFAs) such as butyrate, propionate, and acetate are gut microbial metabolites that are formed from colonic fermentation of dietary fiber. These metabolites maintain gut barrier integrity and are also known to modulate host immunity and pathogen susceptibility (17–20). SCFAs act by inhibiting either class I and IIa histone deacetylases (HDACs) or by binding to free fatty acid receptors (FFAR2 or FFAR3) (17, 18). FFARs belong to the G-protein coupled receptors (GPCR) family, such as GPR43 (FFAR2) and GPR41 (FFAR3). SCFAs act as ligands for FFARs and, upon activation, regulate multiple signaling pathways including ERK1/2, p38, JNK, Akt, intracellular Ca^+2^ release, and inhibition of cAMP accumulation. Recent studies have shown that both SCFAs and their receptors FFARs play a critical role in the regulation of inflammation and adaptive immunity (18–22). They are also known to modulate the pathogenesis of various neurological and ocular diseases (18, 23–27). SCFAs have shown differential roles against invading microbial pathogens, including bacteria and viruses (27–29). Among viruses, SCFA butyrate has been shown to promote the replication of influenza A virus (IAV), human immunodeficiency virus 1 (HIV-1), human metapneumovirus (hMPV), and vesicular stomatitis virus (VSV) (30, 31). In contrast, SCFAs treatment has been suggested to suppress viral replication and associated inflammation in SARS-CoV-2 (22), Herpes simplex virus 1 (HSV-1) (32–34), and hepatitis B virus (HBV) (35) infections. However, the role of SCFAs and their receptor FFAR2 in ZIKV infection and associated inflammatory immune responses has never been demonstrated.

In the present study, we reported the previously unknown role of SCFA derivatives phenylbutyrate (PBA), sodium butyrate (NaB), and sodium acetate (NaAc), and a GPCR-FFAR2 in ZIKV ocular infection. Using synthetic derivatives of butyrate (PBA, NaB) and acetate (NaAc), we show that butyrate and acetate suppressed ZIKV replication and associated ocular manifestations. PBA and NaAc diminished ZIKV-induced inflammatory, interferons (IFNs), and interferon-stimulated gene (ISGs) response. Their activity was mediated via FFAR2, and pharmacological inhibition of FFAR2 significantly enhanced the viral replication, associated cell death, and ocular complications.

## RESULTS

### SCFAs phenylbutyrate and sodium acetate restrict ZIKV replication in HTMC

**Fig 1:**
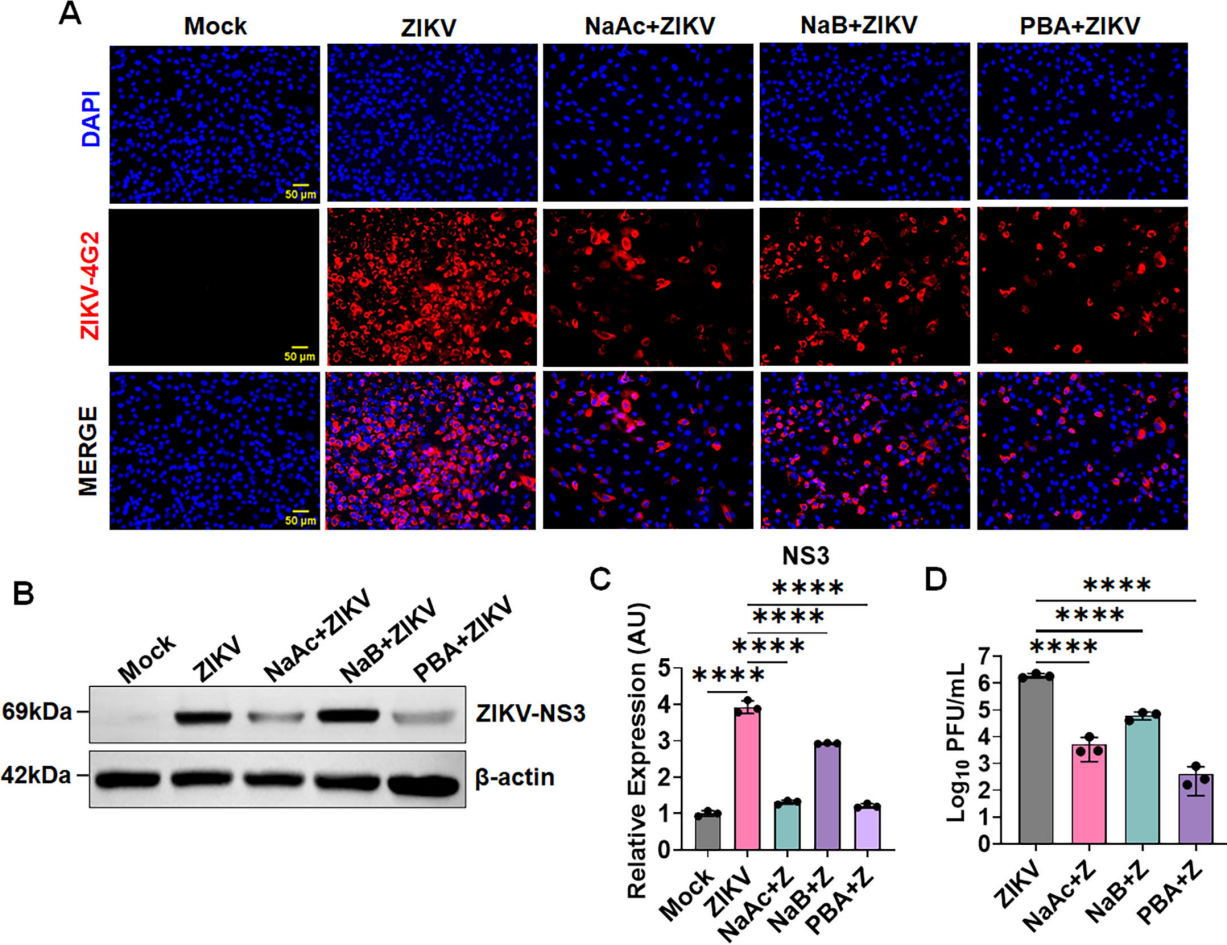
SCFAs restrict ZIKV replication in HTMCs. (**A**) Primary human trabecular meshwork cells (HTMCs, *n* = 3) were pretreated with PBA, NaB, or NaAc followed by ZIKV (Z) (PRVABC59 strain, MOI: 1) infection for 48 h. Mock-treated cells were used as controls. Infected and mock-treated cells were fixed and immunostained for anti-flavivirus E (4G2) antigen. The representative images show the presence of ZIKV (Red) and DAPI-stained nucleus (Blue), scale bar: 50 µm. (**B**) In a second set of experiments, cell lysates from ZIKV-infected, PBA, NaB, NaAc, and mock-treated cells were subjected to immunoblotting for ZIKV nonstructural protein, NS3. (**C**) Densitometric analysis of NS3 immunoblots was performed using ImageJ. The bar graph represents the mean ± SD of three biological replicates. (**D**) The treated and untreated conditioned media were subjected to plaque assay to quantify the replicating virions. The bar graph represents mean ± SD from three biological replicates. ****P  <  0.0001 (one-way ANOVA).

SCFAs butyrate and acetate have demonstrated differential antiviral activities against different viruses. However, their role in flaviviruses, such as ZIKV infection, is entirely unknown. Here, we investigated the role of three SCFAs: PBA, NaB, and NaAc in ZIKV replication using primary human trabecular meshwork cells (HTMCs). Previously, we demonstrated that ZIKV has high tropism toward the anterior segment of the eye and can permissively infect HTMCs (11). To investigate the role of these SCFAs, we first pretreated HTMC with PBA, NaB, and NaAc prior to ZIKV infection. We observed that PBA and NaAc significantly inhibited the ZIKV replication as measured by ZIKV-envelope (E) antigen (4G2) immunofluorescence staining (Fig. 1A). To further confirm the inhibition of ZIKV replication by these SCFAs, we performed immunoblotting for ZIKV nonstructural protein NS3. Our data show that PBA and NaAc significantly inhibited ZIKV replication, as indicated by the suppression of NS3 protein in treated cells compared to untreated cells (Fig. 1B and C). NaB also demonstrated ZIKV inhibitory properties although to a slightly lesser extent than PBA and NaAc (Fig. 1A through C). To further examine the effect of butyrate and acetate on the number of replicating virions, we performed a plaque assay. Our results revealed significant inhibition of ZIKV plaques by PBA, NaB, and NaAc compared to untreated ZIKV infected cells, confirming their anti-ZIKV role (Fig. 1D). Together, our findings confirm that SCFAs phenylbutyrate and sodium acetate can inhibit ZIKV replication and transmission.

### SCFAs butyrate and acetate diminish ZIKV-induced pro-inflammatory cytokines and IFNs/ISGs response

**Fig 2:**
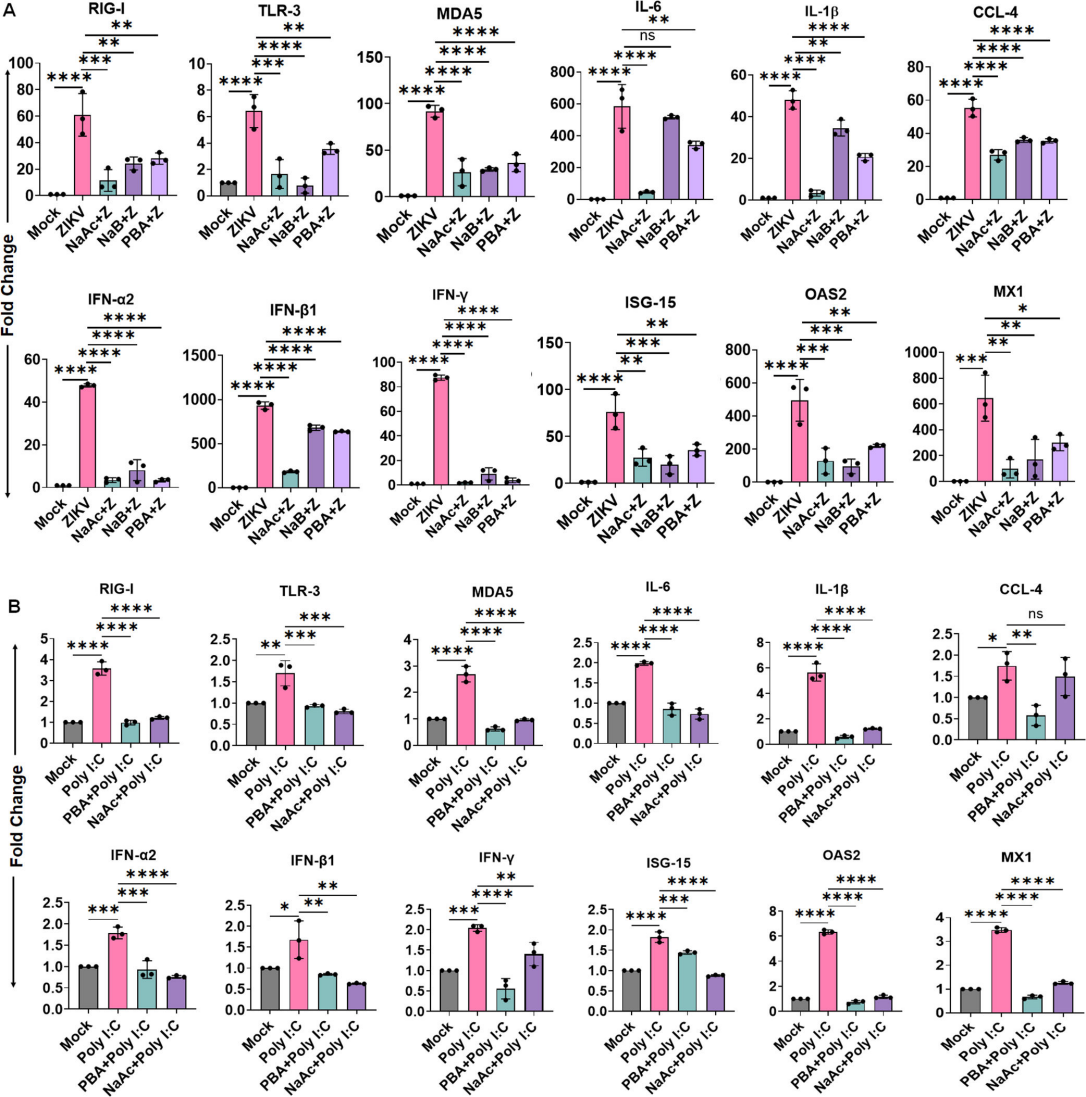
SCFA treatment attenuates ZIKV-induced pro-inflammatory cytokines and IFNs/ISGs response. (**A**) HTMCs (*n* = 3) were pretreated with PBA, NaB, or NaAc followed by ZIKV (Z) (PRVABC59 strain, MOI: 1) infection for 48 h. (**B**) In a second set of experiments, HTMCs (*n* = 3) were pretreated with PBA, NaB, or NaAc and challenged with Poly I:C (100 ng/mL) for 48 h. Mock-treated cells were used as controls. Total RNA extracted from infected and treated cells was subjected to qPCR to quantify the mRNA expression of PRRs (RIG-I, TLR-3, MDA5), inflammatory cytokines/chemokines (IL-6, IL-1β, CCL-4), IFNs (IFN-α2, IFN-β1, IFN-γ), and ISGs (ISG-15, OAS2, MX1) genes. The bar graph represents means ± SD from three biological replicates. *P  <  0.05; **P  <  0.005; ***P  <  0.0005; ****P  <  0.0001; ns: not significant (one-way ANOVA).

SCFAs have been demonstrated to modulate inflammatory and antiviral responses although different studies have shown contentious results. However, how SCFAs modulate inflammatory and antiviral responses in ocular milieu upon ZIKV infection remains unknown. Previously, we demonstrated that ZIKV infection in HTMC induces a dysregulated cytokine and IFN response (11). Thus, to gain molecular insight into the effect of butyrate and acetate on the inflammatory cytokine and IFNs/ISGs response to ZIKV infection, we challenged HTMCs with and without PBA/NaB/NaAc and measured the host innate inflammatory and antiviral response 48 h post-infection via qPCR. Our results show that NaAc, PBA, and NaB significantly reduced the mRNA expression levels for classical pattern recognition receptors (PRRs) (e.g., RIG-I, TLR-3, and MDA5), inflammatory cytokines/chemokines (e.g., IL-6, IL-1β, and CCL-4), IFNs (e.g., IFN-α2, IFN-β1, and IFN-γ), and ISGs (ISG-15, OAS2, and MX1) as compared to ZIKV-infected cells (Fig. 2A).

To further investigate if the observed reduction in cytokines and anti-viral response is due to decreased viral replication, we tested the expression of these mediators in a non-virally stimulated condition. We challenged HTMCs with Poly I:C in the presence and absence of PBA/NaAc and measured the mRNA expression of these PRRs, cytokines, IFNs, and ISGs via qPCR. Similar to ZIKV, PAB and NaAc significantly suppressed the Poly I:C induced PRRs (RIG-I, TLR-3, and MDA5), inflammatory cytokines/chemokines (IL-6, IL-1β, CCL-4), IFNs (IFN-α2, IFN-β1, and IFN-γ), and ISGs (ISG-15, OAS2, and MX1) response (Fig. 2B), indicating the decreased level of innate response is not only due to decreased viral replication.

**Fig 3:**
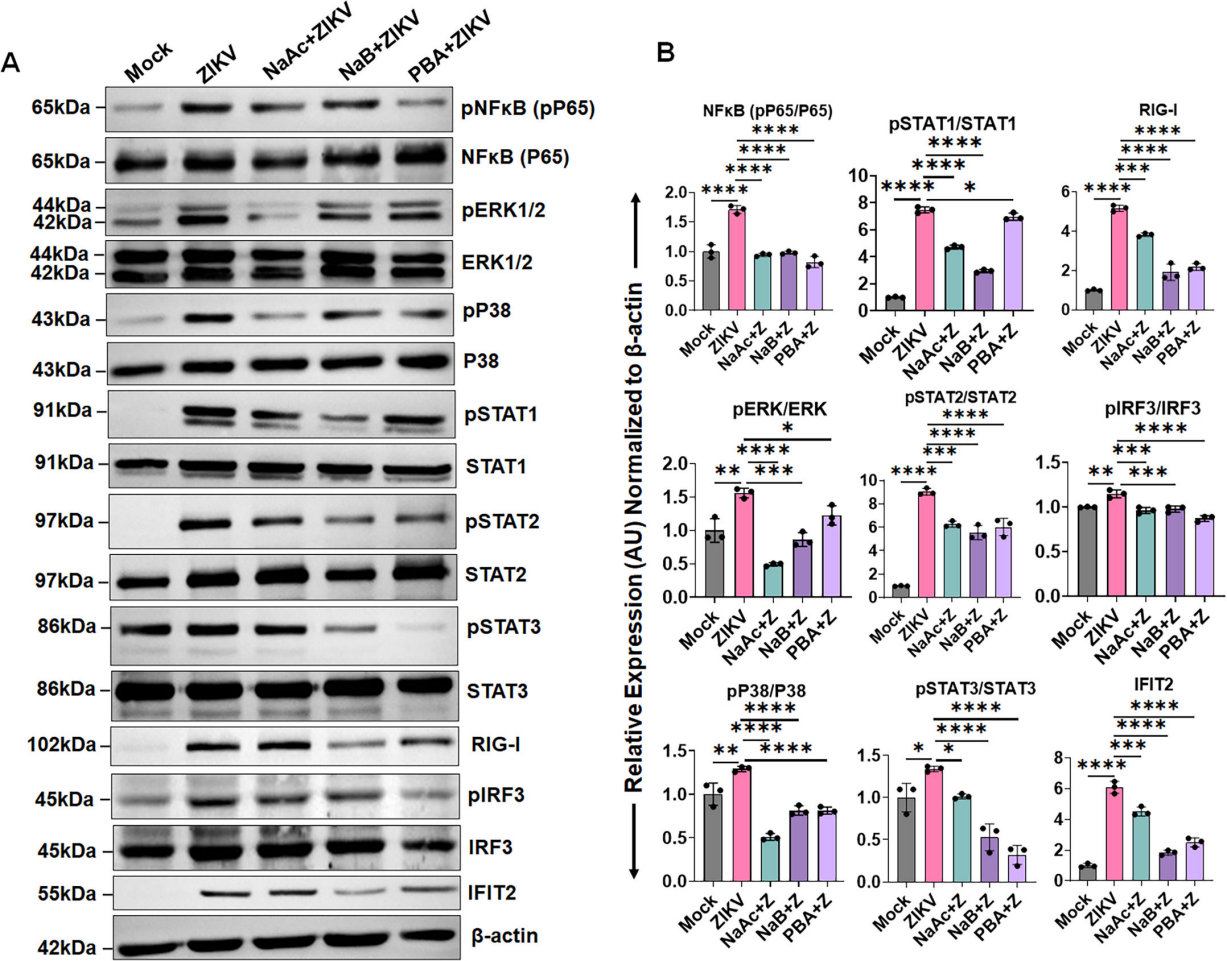
SCFAs antagonize NFκB/MAPK-mediated ISGs signaling. HTMCs (*n* = 3) were pretreated with PBA, NaB, or NaAc followed by ZIKV (Z) (PRVABC59 strain, MOI: 1) infection for 48 h. Mock-treated cells were used as controls. (**A**) The cell lysates from mock, ZIKV-infected, and drug-treated cells were subjected to western blotting for the NFκB, ERK1/2, p38, STAT1, STAT2, STST3, RIG-I, IRF3, and IFIT2 pathways. (**B**) Densitometric analysis of the immunoblots was performed using ImageJ. The bar graph represents means ± SD from three biological replicates. *P  <  0.05; **P  <  0.005; ***P  <  0.0005; ****P  <  0.0001 (one-way ANOVA).

NFκB, MAPK, and STATs signaling regulate the expression of viral-mediated cytokines, IFNs, and ISGs responses. Thus, to identify the mechanism by which NaAc, PBA, and NaB block the expression of these PRRs, cytokines, IFNs, and ISGs, we measured the activation of NFκB, MAPK (ERK1/2 and p38), STAT1, STAT2, and STAT3 signaling pathways in the presence and absence of these SCFAs following ZIKV infection. The western blot analysis of ZIKV-infected HTMC demonstrated increased phosphorylation of NFκB, ERK1/2, p38, STAT1, STAT2, and STAT3 (Fig. 3). In contrast, NaAc, NaB, and PBA treatment significantly inhibited the activation of all of these signaling pathways (Fig. 3). We next assessed whether butyrate and acetate regulated the expression of antiviral/ISGs mediators and found that PBA, NaB, and NaAc treatment suppressed the expression of critical antiviral/ISGs mediators, such as RIG-I, IRF3, and IFIT2 (Fig. 3). Together, our data indicate that SCFAs butyrate and acetate inhibit the expression of pro-inflammatory cytokines, interferons, and ISGs by inhibiting the NFκB, MAPK, and STAT1/2/3 signaling pathways.

### FFAR2/GPCR43 inhibition enhances the HTMC susceptibility toward ZIKV

**Fig 4:**
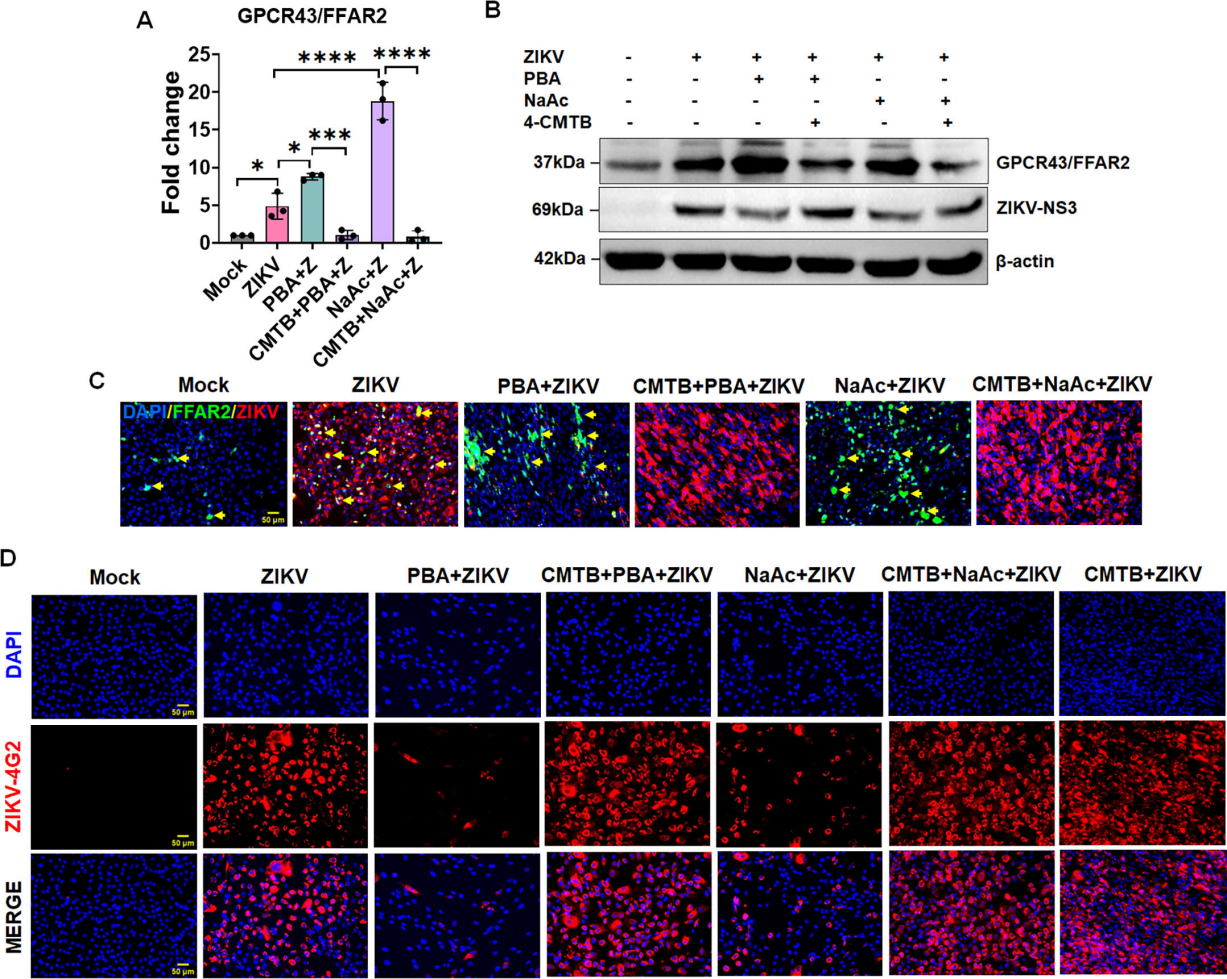
HTMCs express FFAR2, and its inhibition promotes ZIKV replication. HTMCs (*n* = 3) were pretreated (1 h) with FFAR2 inhibitor 4-CMTB, followed by PBA, NaB, or NaAc treatment for another 1 h. After pre-treatment, cells were infected with ZIKV (Z) (PRVABC59 strain, MOI: 1) for 48 h. Mock-treated cells without infection were used as controls. (**A**) Total RNA extracted from mock, ZIKV-infected, and drug-treated cells was subjected to qPCR to quantify the mRNA expression of FFAR2 gene. *P  <  0.05; ***P  <  0.0005; ****P  <  0.0001 (one-way ANOVA). (**B**) From another set of experiments, the cell lysates of infected and treated cells were subjected to western blotting for the FFAR2 and ZIKV NS3 proteins. (**C**) Cells were fixed and co-immunostained for FFAR2 and ZIKV E antigen (4G2) antibodies. The representative images show staining for FFAR2 (Green), ZIKV (Red), and DAPI-stained nucleus (Blue), scale bar: 50 µm. (**D**) In another set of experiments, cells were fixed and immunostained for the presence of ZIKV (Red), DAPI-stained nucleus (Blue), scale bar: 50 µm.

SCFAs, acetate and butyrate, are known ligands for FFAR2/GPCR43. FFAR2 is expressed by the intestinal epithelium, brain, and various subsets of immune cells such as T cells, monocytes, macrophages, neutrophils, and dendritic cells (31, 36). However, their expression by the trabecular meshwork (TM) is unknown. Activation of FFAR2 by SCFAs has been shown to diminish susceptibility toward various microbial pathogens, including bacteria and viruses (37, 38), but their role in flaviviral infections such as ZIKV has never been demonstrated. Therefore, here we aimed to investigate the role of FFAR2 in ZIKV infectivity. To assess the role of FFAR2 in HTMCs, we pretreated the cells with PBA and NaAc prior to ZIKV infection and measured the expression of FFAR2 via qPCR, western blotting, and immunofluorescence staining. Our results from all three assays demonstrated that ZIKV significantly induces the expression of FFAR2 at transcript as well as protein levels in HTMCs, which is potentiated by its ligands PBA and NaAc (Fig. 4A through C). To further test the role of FFAR2 in ZIKV infectivity, we blocked the FFAR2 receptor using a selective pharmacological inhibitor of FFAR2, 2-(4-chlorophenyl)−3-methyl-*N*-(thiazol-2-yl)butanamide (4-CMTB) (31, 39). Our data show that the inhibition of FFAR2 either by 4-CMTB alone or in the presence of PBA/NaAc substantially increased the ZIKV replication in HTMCs, as revealed by increased ZIKV-4G2 immunofluorescence staining (Fig. 4D). Immunoblotting for ZIKV-NS3 protein further confirmed increased viral replication with FFAR2 inhibition (Fig. 4B). The inhibition of the FFAR2 receptor by 4-CMTB was confirmed by qPCR, western blotting, and immunostaining (Fig. 4A through C). Together, our results indicate that PBA and NaAc mediate their antiviral activity via FFAR2 signaling, and inhibition of FFAR2 dramatically enhances the HTMCs susceptibility towards ZIKV infection.

### SCFA treatment suppresses, while FFAR2 inhibition promotes, ZIKV-induced cell death

**Fig 5:**
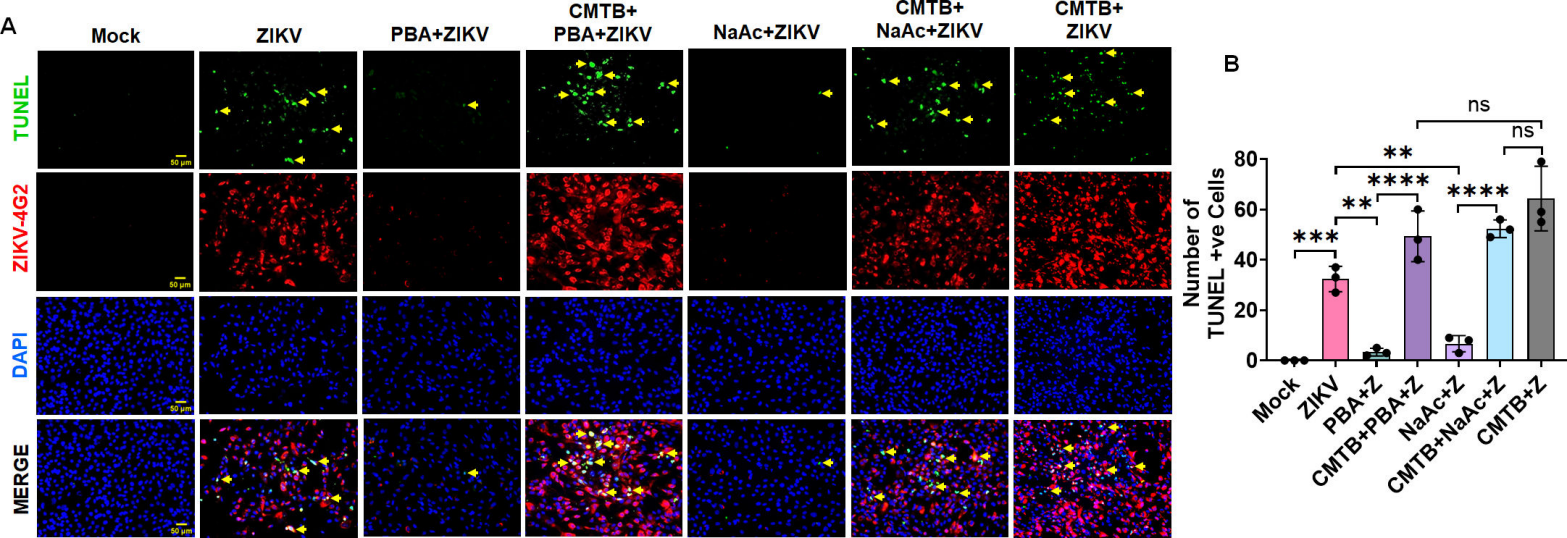
SCFA treatment suppresses, while FFAR2 inhibition promotes, ZIKV-induced cell death. HTMCs (*n* = 3) were pretreated (1 h before) with FFAR2 inhibitor 4-CMTB, followed by PBA, NaB, or NaAc treatment for 1 h. After pre-treatment, cells were infected with ZIKV (Z) (PRVABC59 strain, MOI: 1) for 48 h. Mock-treated cells without infection were used as controls. (**A**) Cells were fixed and subjected to TUNEL assay followed by co-immunostaining for ZIKV E (4G2) antigen. Representative images show TUNEL-positive cells (Green, a few representative marked with yellow arrow) with ZIKV (Red) and DAPI nuclear stain (Blue). Scale bar: 50 µm. (**B**) The average number of TUNEL-positive cells per field (average of 4 frames/slides from *n* = 3) was counted and presented as mean ± SD of TUNEL-positive cells. ***P  <  0.0005; ****P  <  0.0001; ns: not significant (one-way ANOVA).

Previously, we demonstrated that ZIKV induces TM cell death *in vitro* as well as in mouse eyes (11). Here, we sought to test if SCFAs PBA, NaB, and NaAc have any effect on TM cell death and if FFAR2 inhibition modulates ZIKV-induced cell death. To test this, we infected HTMCs with ZIKV in the presence and absence of PBA and NaAc with and without FFAR2 inhibition and performed a TUNEL assay to assess cell death. Our results show that PBA and NaAc treatment significantly protected the HTMCs from ZIKV-induced cell death, as evidenced by decreased TUNEL-positive cells compared to ZIKV-infected/untreated cells (Fig. 5A and B). However, the FFAR2 blocking via 4-CMTB abolished the cell protective ability of PBA and NaAc and significantly enhanced the ZIKV-induced cell death (Fig. 5A and B). Our findings indicate that PBA and NaAc protect HTMCs from ZIKV-induced cell death, and the cell protective ability of these SCFAs is mediated via FFAR2.

### SCFAs inhibit ZIKV infection by direct viral inactivation and impairment of cellular binding and entry

**Fig 6:**
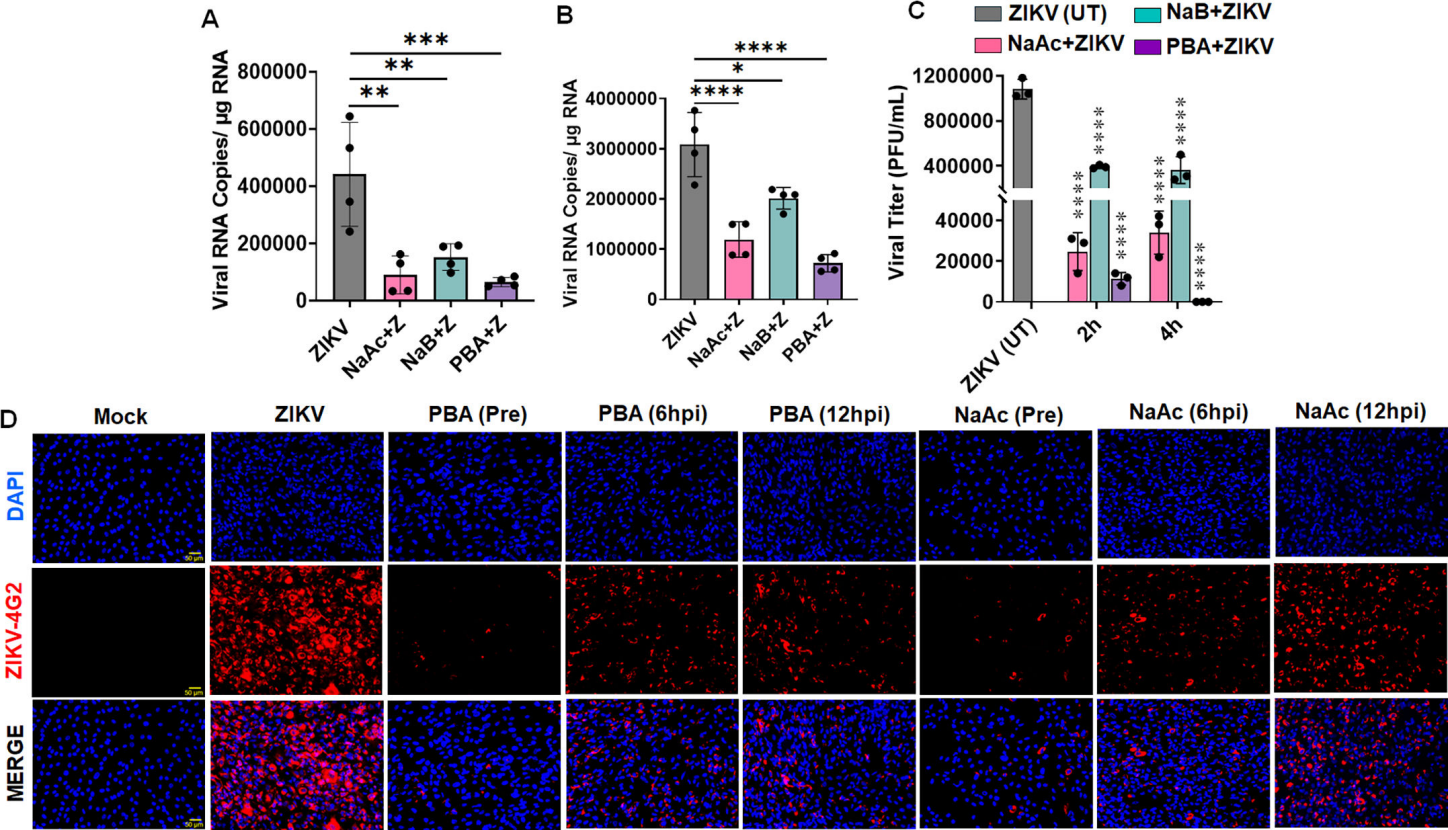
SCFAs inhibit ZIKV infection by directly inactivating the virus and preventing viral attachment and entry into HTMCs. (**A**) HTMCs (*n* = 4) were treated with PBA, NaB, and NaAc for 1  h with incubation at 4°C, followed by ZIKV (MOI: 1) infection. Following incubation at 4°C for 2 h, the total RNA was extracted and subjected to qPCR for the ZIKV envelope gene. The data were presented as mean ± SD of RNA copies/μg RNA. (**B**) HTMCs (*n* = 4) were treated with PBA, NaB, and NaAc for 1  h with incubation at 37°C, followed by ZIKV (MOI: 1) infection. After viral adsorption for 2 h at 37°C, the total RNA was extracted and subjected to qPCR for the ZIKV envelope gene. The data were presented as mean ± SD of RNA copies/μg RNA. (**C**) ZIKV (10^6^ PFU/mL) was incubated with PBA, NaB, or NaAc in a serum-free DMEM at 37°C for 2 h or 4 h. ZIKV incubated without any compound served as the untreated (UT) control. At respective time points, 100 µL of each viral-drug mixture was serially diluted (10^−2^ to 10^−5^), and viral infectivity was determined by plaque assay. The plaques from treated and untreated conditioned media were counted and presented as PFU/mL. (**D**) HTMCs were either pretreated (1 h before) with PBA or NaAc, followed by ZIKV infection, or treated 6 h and 12 h post-ZIKV infection. Mock-treated and ZIKV-infected cells without treatment were used as controls. Forty-eight hours after infection, cells were fixed and immunostained for ZIKV E (4G2) antigen. The representative images show the presence of ZIKV (Red) and DAPI-stained nucleus (Blue), scale bar: 50 µm. *P  <  0.05; **P  <  0.005; ***P  <  0.0005; ****P  <  0.0001 (one-way ANOVA).

SCFAs have shown differential antiviral activities against different viruses. Some studies have shown that SCFAs supports viral replication by suppressing the interferon response, while some have suggested suppressed viral replication despite the reduction of inflammatory and antiviral response (30, 40). We were puzzled by our observations that butyrate and acetate inhibit ZIKV replication in HTMCs as well as suppress the inflammatory/IFNs/ISGs response. This indicates an alternative antiviral mechanism of SCFAs independent of IFNs/ISGs signaling. Thus, we aimed to determine the mode of action of these SCFAs on ZIKV. SCFAs have been shown to influence viral entry, replication, and reactivation (31, 41, 42). Therefore, we decided to test whether SCFAs, butyrate and acetate, alter ZIKV binding and entry to HTMCs. To test this, we performed viral attachment and entry assays by measuring the viral RNA copy numbers following incubation with SCFAs as described in the methodology section. Our results from the viral attachment assay revealed a significant decrease in viral copy numbers with SCFAs treatment (Fig. 6A). Similarly, the entry assay also demonstrated a substantial decline in viral copy numbers (Fig. 6B), confirming the ability of butyrate and acetate to modulate viral internalization. To assess the direct virucidal effect of SCFAs, ZIKV was incubated with PBA, NaB, or NaAc in a cell-free medium for 2 and 4 h. Following incubation, the drug-viral mixtures were serially diluted (2-5 log), and the viral infectivity was quantified by plaque assay. Our data revealed that all three SCFAs significantly reduced the number of plaques (> 2 log_10_) compared to untreated control, indicating viral inactivation (Fig. 6C).

To further confirm the effect of SCFAs on viral internalization, we tested the anti-ZIKV activity of PBA and NaAc in pre-infection (before viral internalization) and 6 and 12 h post-infection (after viral internalization) treatment settings. We observed that PBA and NaAc suppressed the viral replication in pretreated groups; however, ZIKV positivity was considerably higher in post-treatment settings in comparison to the pretreated groups (Fig. 6D). Though, both pre- and post-treatment with butyrate and acetate suppressed the viral replication up to some extent compared to ZIKV-infected and untreated cells (Fig. 6D). Collectively, these results indicate that SCFAs can manipulate early viral life cycle by modulating viral attachment and entry to the HTMCs.

### SCFA treatment attenuates, while FFAR2 inhibition exacerbates, ZIKV-induced ocular manifestations in mice

**Fig 7:**
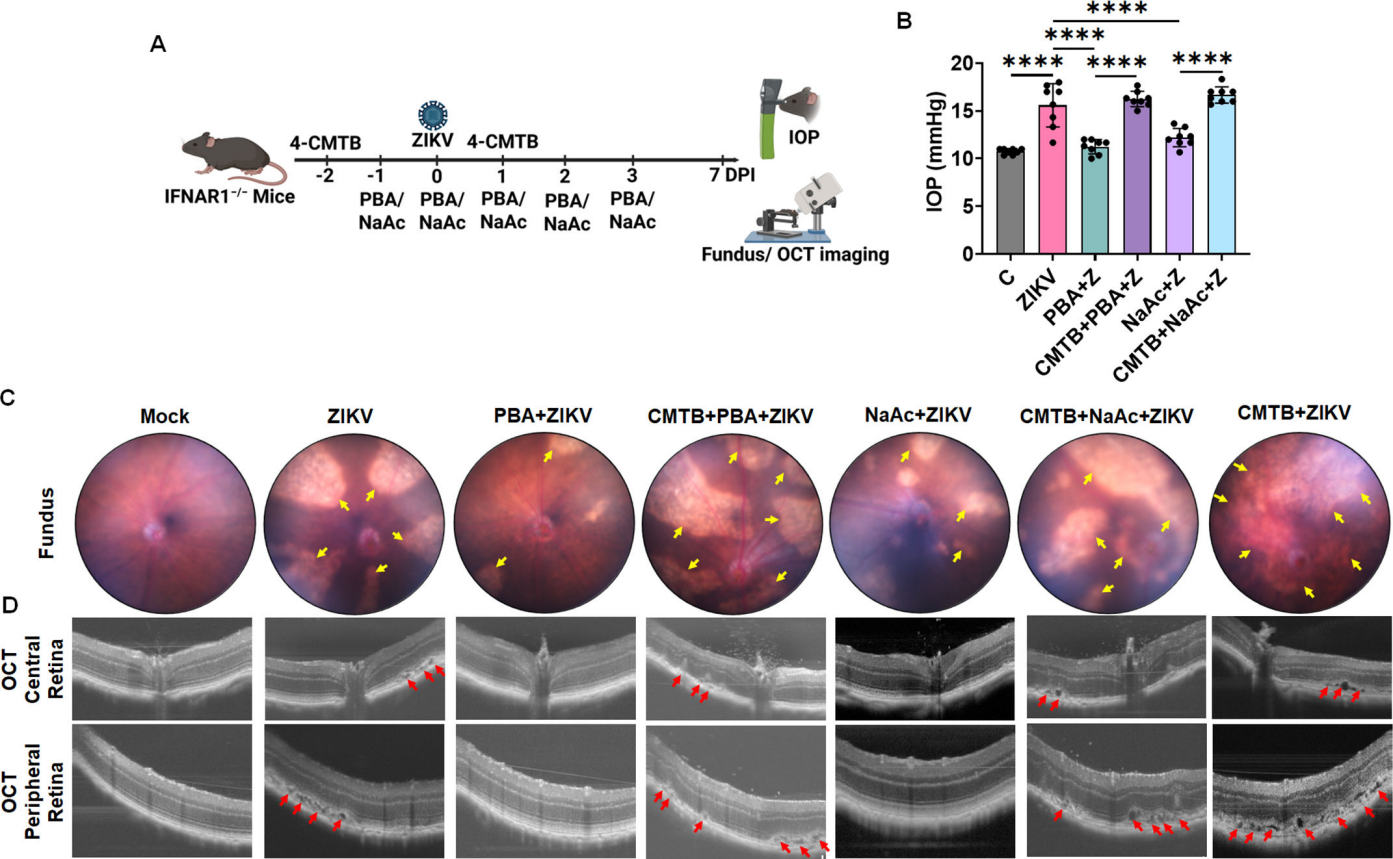
SCFA treatment attenuates, while FFAR2 inhibition aggravates, ZIKV-induced ocular pathology in mice. IFNAR1^−/−^ mice (*n* = 6–8) were pretreated with FFAR2 inhibitor 4-CMTB (days −2 and 1), followed by PBA, NaB, or NaAc (days −1 to 3 days post infection [DPI]) via i.p. administration. Following treatment, mice were infected with ZIKV (1 × 10^4^ PFU) via intracameral injection. Mice with saline injection without ZIKV infection were used as mock controls. (**A**) Timeline for drug administration and ZIKV infection. (**B**) At 7 DPI, mouse IOP were recorded and represented as mean IOP ± SD. (**C**) Fundus and (**D**) OCT imaging were performed to determine the retinal manifestations using the Micron IV fundus camera and image-guided OCT2 system. Representative funduscopic and OCT micrographs show RPE and chorioretinal atrophy (shown with yellow arrows [on fundus images, panel **C**], with RPE and outer retinal layer disruption [shown with red arrows (on OCT images, **D**) in treated/untreated groups. The timeline shown in panel A was created using BioRender software (biorender.com).

Previously, we showed that ZIKV induces anterior segment (AS) inflammation leading to TM damage and elevated intraocular pressure (11). We also demonstrated that ZIKV migrates from the anterior chamber to the back of the eye and causes chorioretinal atrophy, retinal and optic nerve damage (10, 11). After observing the anti-ZIKV effect *in vitro* in HTMCs, we reasoned to test their therapeutic potential against ZIKV-induced ocular complications in mice. To test this, we pretreated (1 day before) mice with PBA and NaAc via i.p. administration, followed by ZIKV infection and subsequent treatment for 4 consecutive days. For FFAR2 inhibition groups, we pretreated mice with 4-CMTB via i.p. injections 1 day before PBA/NaAc pre-treatment and once again on the 3^rd^ day after the first administration for consistent FFAR2 blocking. To test the baseline effect of FFAR2 inhibition on ZIKV-induced ocular pathology, we treated another group of animals with 4-CMTB alone without any SCFAs treatment. The timeline for the 4-CMTB/SCFAs treatment and ZIKV infection is shown in Fig. 7A. As anticipated, ZIKV infection significantly elevated the IOP (Fig. 7B), caused severe RPE/chorioretinal atrophy (Fig. 7C), and RPE/outer retinal layer disruption (Fig. 7D) in comparison to uninfected controls. In contrast, PBA and NaAc treatment significantly reduced the IOP (Fig. 7B) and attenuated the ZIKV-induced RPE/retinal damage as revealed by fundus (Fig. 7C) and optical coherence tomography (OCT) (Fig. 7D) imaging. Remarkably, pharmacological inhibition of FFAR2 by 4-CMTB antagonizes the protective ability of PBA/NaAc and aggravates ZIKV-induced RPE/chorioretinal atrophy, outer retinal layer disruption, and IOP elevation (Fig. 7B through D). The fundus and OCT exam on the 4-CMTB alone-treated group showed dramatically enhanced ZIKV-induced pathology compared to the rest of the treatment groups (Fig. 7C and D).

**Fig 8:**
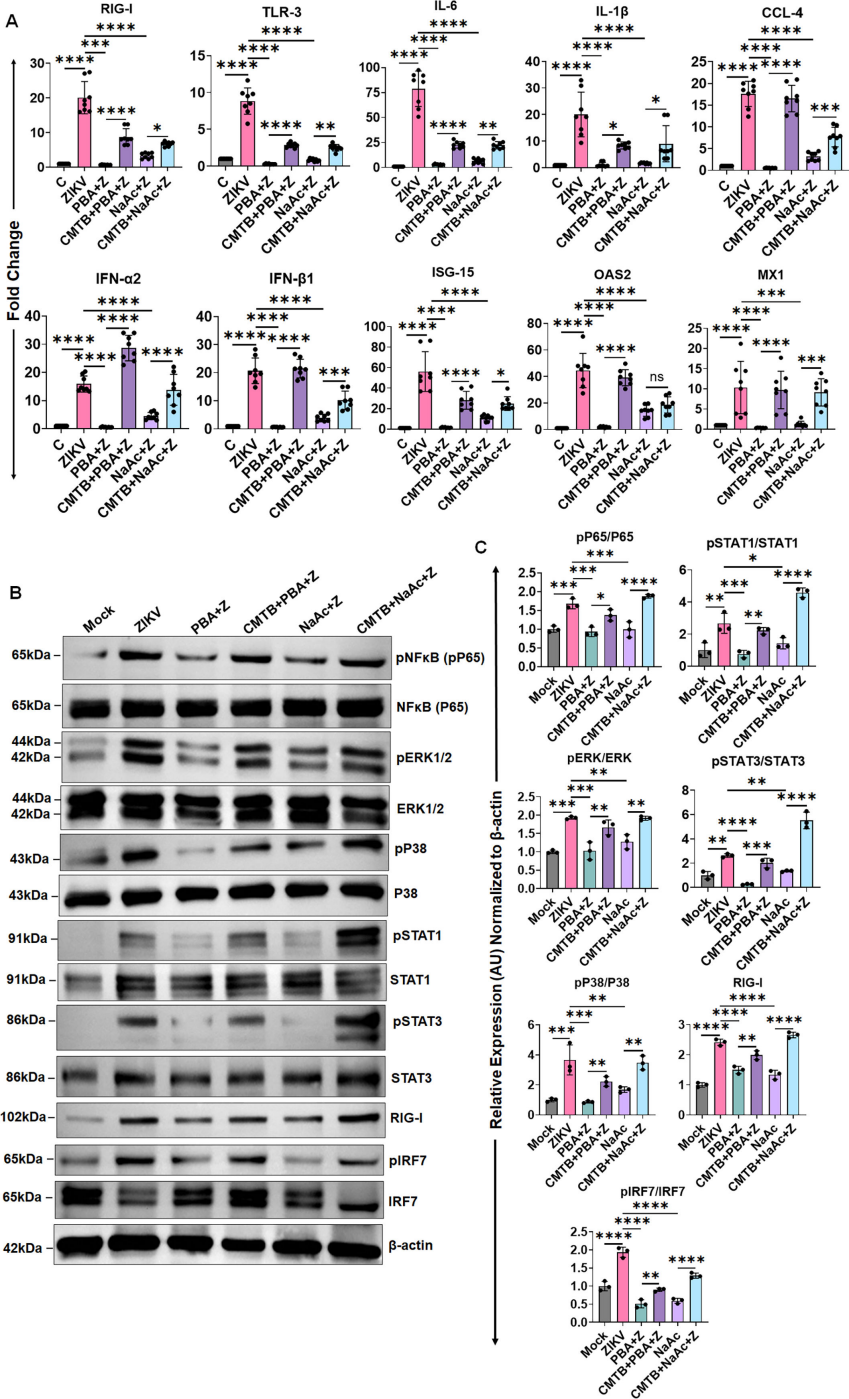
SCFA treatment diminishes, while FFAR2 inhibition exacerbates, ZIKV-induced mouse anterior segment’s inflammatory response. IFNAR1^−/−^ mice (*n* = 6–8) were pretreated with FFAR2 inhibitor 4-CMTB followed by PBA, NaB, or NaAc via i.p. administration, and ZIKV infection as per the timeline shown in Fig. 7A. Seven days post-infection, anterior segment/TM tissue from treated and untreated mice were harvested and subjected to (**A**) RNA extraction and qPCR to measure the mRNA expression levels of PRRs (RIG-I, TLR-3), inflammatory (IL-6, IL-1β, CCL-4), IFNs (IFN-α2, IFN-β1), and ISGs (ISG-15, OAS2, MX1), and (**B**) western blot for NFκB, MAPKs (ERK1/2, P38), STAT1, STAT3, RIG-I, and IRF7 inflammatory/ISG pathways. (**C**) Densitometric analysis of the immunoblots was performed using ImageJ. *P  <  0.05; **P  <  0.005; ***P  <  0.0005; ****P  <  0.0001 (one-way ANOVA).

To further test the role of PBA and NaAc and their receptor FFAR2 on ZIKV-induced AS inflammation, we measured the expression of various inflammatory mediators in the AS tissue via qPCR with and without FFAR2 inhibition. Our result reveals that butyrate and acetate treatment significantly diminished the mRNA expression of ZIKV-induced PRRs (RIG-I, TLR3), inflammatory cytokines/chemokines (IL-6, IL-1β, CCL-4), IFNs (IFN-α2, IFN-β1), and ISGs (ISG-15, OAS2, MX1) in mouse AS tissue (Fig. 8A). Blocking the FFAR2 receptor by 4-CMTB reversed the anti-inflammatory effect of PBA and NaAc and significantly enhanced the expression of these inflammatory mediators. Since we observed an antagonizing effect on inflammatory and ISGs pathways by PBA and NaAc *in vitro*, we tested the role of these SCFAs on these pathways in our *in vivo* model. Similar to HTMCs, PBA/NaAc inhibited the NFκB, MAPKs (p38, ERK1/2), and STATs (STAT1, STAT3) and ISGs (RIG-I, IRF7) pathways activation in mouse AS/TM tissue, while FFAR2 inhibition reversed these inhibitory properties (Fig. 8B and C). Together, these findings indicate that SCFA suppresses ZIKV-induced AS inflammation and ocular manifestations, and the *in vivo* protective effect of acetate and butyrate is also mediated via FFAR2.

## DISCUSSION

Recent advancements in the microbiome-immunity axis have uncovered the involvement of gut microbiomes and their metabolites in multiple diseases. A recent study demonstrated alterations in gut microbiota in immunocompetent mice (43) and enhanced peripheral and nervous system inflammation due to decreased SCFA levels in macaques (44) during ZIKV infection. SCFAs, acetate, butyrate, and propionate have demonstrated promising therapeutic potential against multiple microbial pathogens, including bacteria and viruses. Although due to both pro- and anti-viral properties, SCFAs have shown very complex and contrary roles with different viruses (17). Emerging literature suggests an interplay between gut microbiota and ocular health (45, 46). However, the role of SCFAs against ZIKV ocular infection remains unknown. Here, we showed a previously unreported role of butyrate and acetate and their receptor FFAR2 in ZIKV ocular pathogenesis. In this study, we discovered that SCFAs, PBA, NaB, and NaAc restrict ZIKV transmission and modulate viral attachment and internalization to the cells. We further uncovered that PBA and NaAc mediate their anti-ZIKV activity via FFAR2, and pharmacological inhibition of FFAR2 antagonizes the protective abilities of PBA and NaAc. SCFA treatment attenuates ZIKV-induced pro-inflammatory response, cell death, and ocular complications.

ZIKV infection is linked to multiple congenital malformations, including microcephaly and ocular manifestations. *In utero* exposure to ZIKV caused severe ocular abnormalities, including chorioretinal atrophy, RPE mottling, optic nerve damage, and congenital glaucoma (10, 14–16). Trabecular meshwork (TM) regulates the intraocular pressure (IOP), and pathologic stress may cause TM damage, resulting in increased glaucoma phenotype (11, 47). Recently, we discovered that ZIKV has high tropism toward the anterior segment of the eye and can cause TM damage, resulting in increased IOP and retinal ganglion cell (RGC) loss (11). In this study, we aimed to test whether SCFAs can protect TM from ZIKV infection. We found that SCFA derivatives PBA, NaB, and NaAc dramatically reduced ZIKV replication and transmission. We further discovered that butyrate and acetate inhibit viral binding and cellular entry to the TM cells. The SCFAs also protected TM from ZIKV-induced cell death. Our study corroborated with previous findings where SCFA treatment has been shown to suppress replication of SARS-CoV-2 (22), HBV (35), HSV-1 (32–34), and porcine epidemic diarrhea virus (PEDV) (48), and exacerbate disease severity. Similarly, butyrate has been shown to effectively reduce rotavirus-induced cell death and provide protection against intestinal epithelial barrier damage (42, 49). In contrast to these findings, a few other studies have shown that butyrate could promote the replication of EBV (50), IAV, HIV-1, hMPV, VSV (30), and TGEV (51), whereas it has no effect on SeV replication (30). These findings suggest that, given the diverse role of SCFAs among different viruses, it is imperative to interpret their pro- or anti-viral activity within specific relevant physiological contexts.

Upon viral infection, host cells induce a pro-inflammatory and IFN response, which plays a crucial role in curbing infection. However, uncontrolled immune activation upon infection may lead to persistent inflammation, resulting in tissue damage, morbidity, and mortality. The recurrent inflammation of ocular tissue, an immune-privileged organ, is detrimental and results in vision loss. ZIKV has been shown to induce inflammatory cytokines, IFNs, and ISGs in different models (52). We recently observed a dysregulated immune response via ZIKV in TM, resulting in trabeculitis and TM damage (11). SCFAs are well-characterized for their anti-inflammatory activities via HDAC inhibition (17, 51). In addition, PBA is also a well-known endoplasmic reticulum stress inhibitor and has been shown to modulate CREB3 pathways in HSV-induced ocular infection, suppress viral replication, and associated inflammation (32). Recent studies have demonstrated that SCFAs can suppress inflammatory cytokines and virus-induced IFNs/ISGs response to confer protection from tissue damage (17, 33, 34). In line with these studies, we observed that PBA, NAB, and NaAc can suppress ZIKV-induced inflammatory (IL-6, IL-1β, CCL-4), IFNs (IFN-α2, IFN-β1, IFN-γ), and ISGs (ISG-15, OAS2, MX1) responses in TM. During RNA virus infection, the inflammatory and antiviral immune response is initiated upon PRRs (RIG-I, MDA5, TLR-3), followed by activation of NFκB/MAPKs/STATs pathways. ZIKV activates RIG-I, MDA5, and TLR-3 receptors and mediates innate immune response via NFκB/MAPK pathways (53, 54). SCFA treatments have been shown to antagonize the NFκB pathway upon HSV-1 (34) and Japanese encephalitis virus (55) infection. In this study, we observed that PBA, NaB, and NaAc suppressed the activation of PRRs (RIG-I, TLR-3, MDA5), NFκB, MAPKs (ERK1/2, p38), and IFNs/ISGs mediators, including STATs (STAT1, STAT2, STAT3), IRF3, and IFIT2. Similar to our findings, butyrate has shown inhibition of IFNβ-induced RIG-I and IFIT2 expression (30). In contrast, this study reported that butyrate has no effect on STAT1 and STAT2 upon IFN-β stimulation. This study also concluded that butyrate has a differential role in ISG expression; it suppressed 60% of IFN-induced ISGs while upregulating 3% of IFN-induced ISGs (30). Another study by He et al. reported increased IFN and ISG production by butyrate against PEDV in porcine intestinal epithelial cells (48). Similarly, acetate has shown enhanced IFN-β production against RSV (38) and NLRP3-IFN-I-mediated antiviral response against IAV (56). However, our finding corroborated with other studies showing the suppression of JAK/STAT pathways in LPS-stimulated corneal fibroblasts (57) and RIG-I signaling in TGEV and SARS-CoV-2 infection models (51, 58). These differential effects could be potentially due to either the stressor (IFN-β vs PEDV vs ZIKV infection) or the cell models (transformed colon/lung cell line vs porcine epithelial cells vs human primary TM) used in these studies, vs our study. As discussed by Yin et al., the differential effect of SCFAs could also be attributed to the distinct SCFAs treatment methods (51). Indeed, we observed alteration of viral binding and cellular entry by SCFAs and, therefore, differential effects in viral dissemination in pre- vs post-treatment. Similarly, one other study has demonstrated the differential impact of acetate treatment in mice with simultaneous, before, or after IAV infection (56). Collectively, these findings imply the pleiotropic role of SCFAs on viral infection and immune function and underline careful consideration when applying the findings to human patients with different viral infections.

Canonically, SCFAs mediate their activity by activating the GPCRs, also known as the free fatty acid receptors (FFAR). FFAR2 is expressed by multiple cells, including immune cells, skeletal muscles, smooth muscles, and neurons, and, therefore, assists in maintaining homeostasis by SCFAs in the colon, kidney, joints, lungs, and brain. In the eye, FFAR2 is expressed by corneal epithelium and endothelium, iris, ciliary body, inner nuclear, outer nuclear, and ganglion cell layers (18). FFAR2 plays a critical role in regulating gut homeostasis, energy utilization, immune cell functions, and suppression of inflammatory response. It has also been shown to play a protective role in non-infectious ocular diseases such as uveitis (18). However, their role in ZIKV ocular pathogenesis is entirely unknown. Here, we observed for the first time that TM cells express the FFAR2 receptor and ZIKV modulates its expression. Our study revealed that butyrate and acetate potentiated the FFAR2 expression on TM, and pharmacological inhibition of FFAR2 promoted ZIKV replication. Furthermore, FFAR2 inhibition enhanced the ZIKV-induced cell death, which was suppressed by PBA and NaAc. Our findings corroborated with previous studies where FFAR2 knockdown has shown enhanced susceptibility to *C. rodentium (59*), *Klebsiella pneumoniae* lung infection (60), and TLR-induced keratitis (18). Activation of FFAR2 in pulmonary epithelial cells has shown reduced virus-induced cytotoxicity (38). Similarly, targeted activation of FFAR2 has shown diminished susceptibility toward *S. aureus*, RSV, and IAV and *Streptococcus pneumoniae* superinfection (28, 37, 38, 61).

**Fig 9:**
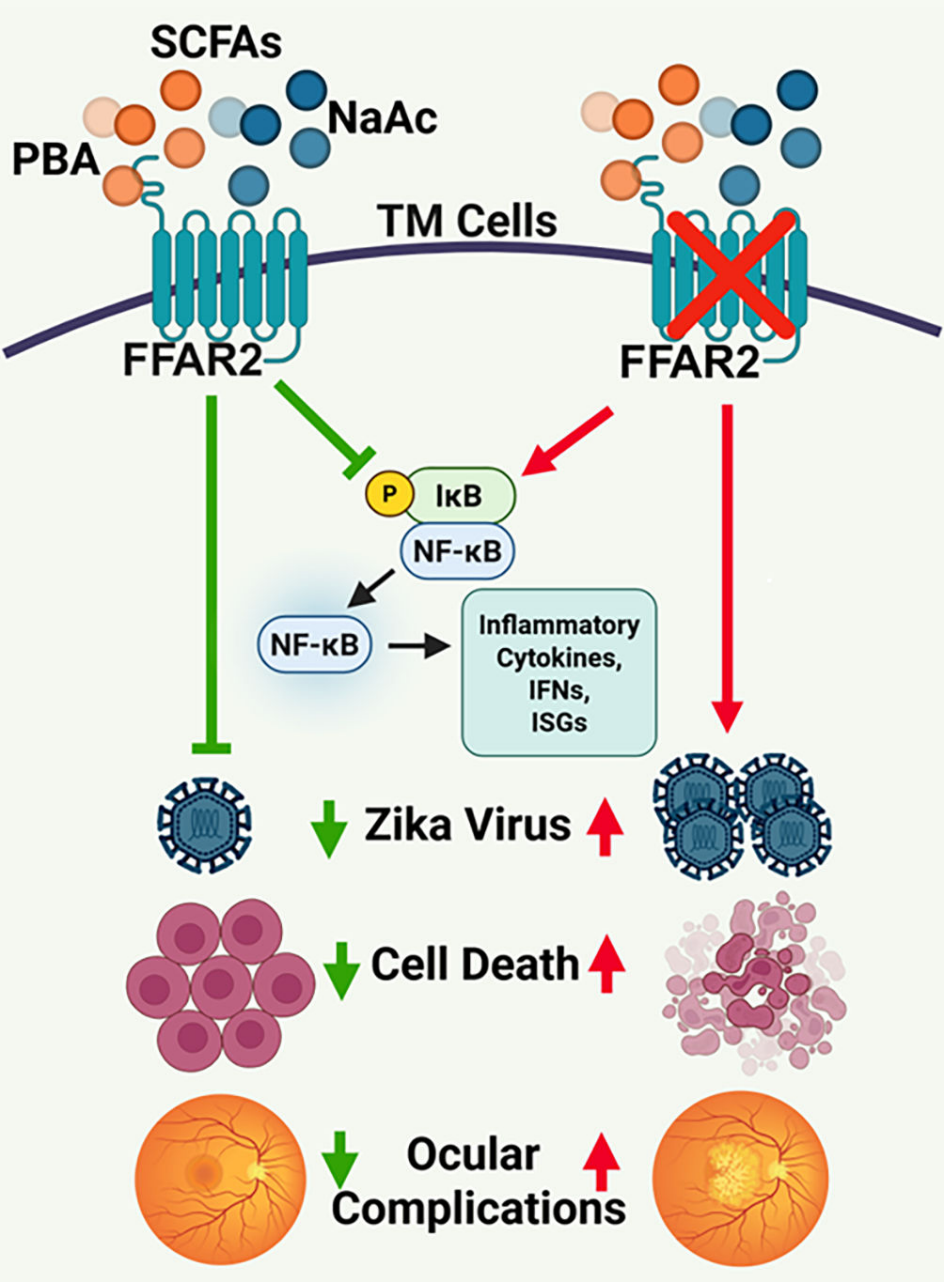
SCFAs butyrate and acetate exhibit anti-ZIKV effects via FFAR2 signaling. Butyrate and acetate suppress ZIKV replication and transmission and reduce associated ocular manifestations. The anti-ZIKV effects of these SCFAs are mediated through FFAR2 signaling, as pharmacological inhibition of FFAR2 enhances ZIKV replication, promotes cell death, and exacerbates ZIKV-induced ocular damage. The schematic diagram was created using BioRender software (biorender.com).

In summary, our work reveals an antiviral role of SCFAs, butyrate, and acetate, restricting ZIKV transmission and associated ocular manifestations via FFAR2 signaling (Fig. 9). The inhibition of FFAR2 reverts the protective effect of butyrate and acetate and promotes ZIKV-induced cell death. Mechanistically, butyrate and acetate not only directly inactivate ZIKV in a cell-free environment but also inhibit viral binding and cellular entry into the HTMCs. Collectively, our findings, along with those of others, indicate that SCFAs have multiprong effects; they can modulate the host responses as well as viral infection by interfering with early stages of the viral lifecycle, including viral binding and entry to the cells. Our study could aid the future design of therapeutic interventions to treat or prevent ZIKV infection and associated ocular complications in humans. However, given the diverse effects of SCFAs on different viruses, their safety and efficacy must be ensured prior to therapeutic use in human subjects with various viral infections. Although we demonstrated the role of FFAR2 in ZIKV transmission using a potent and selective inhibitor of FFAR2, 4-CMTB, future studies using FFAR2 knockout are essential to decipher the role of FFAR2 in ZIKV pathogenesis. Similarly, dietary manipulations or appropriate supplements to augment SCFA production could be explored as potential antiviral strategies against ocular viral infection.

## MATERIALS AND METHODS

### Antibodies and reagents

Antibodies used in this study were purchased from the following sources: 4G2 (GeneTex, #GTX57154), NS3 (GeneTex, #GTX133309), β-actin (Millipore Sigma, #A2228), pNFκB (#3033), NFκB (#6956) pERK1/2 (#4370), ERK (#4695), pP38 (#4511), P38 (#8690), pSTAT1 (#9167), STAT1 (#14994), pSTAT2 (#88410), STAT2 (#72604), pSTAT3 (#9145), STAT3 (#9139), RIG-I (#3743), pIRF3 (#79945), IRF3 (#4302), and IFIT2 (#92633) antibodies were purchased from Cell Signaling Technology (Danvers, MA). SCFAs NaAc (Thermofisher, #A13184-30), 4-PBA (#11323), NaB (#13121), and FFAR2 receptor antagonist 4-CMTB (#29680) were purchased from Cayman Chemicals (Ann Arbor, MI). Poly I:C (#tlrl-pic) was purchased from Invivogen (San Diego, CA).

### Cell culture

Primary human trabecular meshwork cells (HTMCs) (11) were cultured in Dulbecco’s minimal essential medium (DMEM) low glucose GlutaMAX medium, supplemented with 10% fetal bovine serum (FBS), 1× penicillin-streptomycin solution in a CO_2_ (5%) incubator at 37°C. Vero E6 cells (ATCC CRL-1586) were cultured in DMEM low glucose GlutaMAX medium containing 1× penicillin-streptomycin supplemented with 10% FBS. For virus propagation, Aedes albopictus, C6/36 cells (ATCC CRL-1660) were cultured in Eagle’s Minimum Essential Medium (EMEM) containing 1× penicillin-streptomycin supplemented with 10% FBS in a CO_2_ incubator at 30°C.

### Zika virus strain and infection procedure

The ZIKV strain PRVABC59 (NR-50240) was obtained from BEI Resources, National Institute of Allergy and Infectious Diseases (NIAID), and propagated in Aedes albopictus, C6/36 cells. The viral titers were determined using the plaque assay. Small aliquots were prepared and stored in a −80°C freezer for infection studies.

For *in vitro* experiments, HTMCs were challenged with ZIKV at a multiplicity of infection (MOI) of 1. For pharmacological inhibition/activation studies, cells were pretreated with respective drugs/antagonists for 1 h, followed by virus adsorption in serum-free media for 1 h, until indicated otherwise. After adsorption, cells were replenished with media containing drugs and incubated until the desired endpoint. For Poly I:C challenge studies, HTMCs were treated with Poly I:C (100 ng/mL) for 48 h.

### Mice

IFNAR1^−/−^ mice (B6 background, MMRRC Strain # 032045-JAX) were originally purchased from Jackson Laboratories and bred in-house in a germ-free University of Missouri (MU) Office of Animal Resources (OAR) facility. Mice aged 6–10 weeks (male and female) were used in this study. Animals were housed in a restricted-access AAALAC-accredited animal facility. All animals were maintained in a 12:12 h light/dark cycle, with free access to food (rodent chow, Labdiet Pico Laboratory, Saint Louis, MO) and water.

### Mouse infection and SCFA treatment

For SCFA treatment, mice were pretreated with PBA/NaAc (100 mg/kg) via i.p injections 1 day prior to infection and 3 consecutive days post-ZIKV infection. In the FFAR2 inhibition groups, mice were pretreated with 4-CMTB (10 mg/kg) via i.p. injection 1 day prior to PBA/NaAc treatment and another dose on day 3 (39) since the first administration as per the timeline shown in Fig. 7A. For the ZIKV infection, anesthetized animals were inoculated with 1 × 10^4^ PFU of ZIKV via intracameral injections as described previously (11). Seven days post-infection, IOP was recorded using a Tonolab tonometer, and fundus imaging was performed using Micron IV (Phoenix-Micron Inc., Bend, OR). Animals were euthanized, and anterior segment tissue was harvested for inflammatory cytokines/pathways assessment via qPCR and immunoblotting.

### Viral attachment, entry, and inactivation assay

The viral attachment and entry assay was performed as described previously (62). Briefly, HTMCs were pre-incubated with PBA (3 mM), or NaB (3 mM), or NaAc (200 µM) at 4°C (for attachment assay) or 37°C (for entry assay) for 1 h, followed by ZIKV infection (MOI:1). After viral adsorption for 2  h, the cells were washed three times with fresh DMEM media, and total RNA was isolated. The viral RNA copy numbers were determined from whole-cell RNA using a TaqMan probe against the ZIKV envelope (E) gene via qPCR.

For the direct viral inactivation assay, ZIKV (1 × 10^6^ PFU/mL) was incubated with PBA (3 mM), or NaB (3 mM), or NaAc (200 µM) in a cell-free medium (serum-free DMEM) at 37°C for 2 or 4  h. ZIKV incubated without any drug was used as an untreated control. After incubation, 100 µL of each viral-drug mixture was serially diluted (10^−2^ to 10^−5^ fold), and viral infectivity was determined by plaque assay.

### Plaque assay

ZIKV plaque assay was performed using a protocol we described recently (2). Briefly, a confluent monolayer of Vero cells was infected with serial dilutions of ZIKV stock culture. One hour following viral adsorption, the cell monolayer was overlayed with the first overlay media containing a 1:1 mixture of 2× EMEM, 4% FBS, 2× P/S, 20 mM MgCl_2_, and 1.6% Noble Agar. The following day, a second overlay media containing DMEM, 1 mg/mL BSA, 40 mM MgCl_2_, 0.2% glucose, 2 mM sodium pyruvate, 4 mM L-glutamine, 4 mM oxaloacetic acid, 1× P/S, and 0.1% sodium bicarbonate was added. The plates were incubated at 37°C for 5 days in a CO_2_ incubator. Following incubation, cells were fixed with 10% Tricarboxylic Acid (TCA) for 20 min, and the agar overlay was removed gently without disturbing the cell monolayer. Viral plaques were stained with 0.2% crystal violet for 20 min, followed by a wash with MilliQ water. The plaques were counted, and titers were estimated as log_10_ PFU/mL.

### Immunofluorescence assay

For IFA, cells were seeded in a Nunc four-well chamber slide (Fisher Scientific, Rochester, NY) and infected with ZIKV at an MOI of 1 at ~70%–80% confluency. Mock-treated/uninfected cells were used as controls. At desired endpoints, cells were fixed using 4% paraformaldehyde for 10 min at room temperature (RT). After three washes with 1× PBS, cells were blocked and permeabilized using 1% (wt/vol) BSA with 0.4% (vol/vol) Triton X-100 made in PBS (blocking buffer) for 1 h at RT in a humidified chamber. Cells were then incubated with the primary mouse/rabbit antibodies in the blocking buffer (1:100 dilution) overnight at 4°C in a humidified chamber. Subsequently, after three washes with 1× PBS, cells were incubated with anti-mouse/rabbit Alexa Fluor 488/594-conjugated secondary antibodies (1:200 dilutions) for 1 h at RT. Finally, cells were washed four times with 1× PBS and mounted in Vectashield anti-fade mounting medium containing DAPI (Vector Laboratories, Burlingame, CA). The slides were visualized and imaged using a Keyence (Keyence, Itasca, IL) fluorescence microscope.

### TUNEL assay

The cell death was estimated using the TUNEL assay (63). HTMCs were grown in Nunc four-well chamber slides (Fisher Scientific, Rochester, NY) and infected with ZIKV in the presence or absence of SCFAs or 4-CMTB. TUNEL assay was performed using ApopTag Fluorescein *In Situ* Apoptosis Detection Kit (#S7110) per the manufacturer’s instructions (Millipore Sigma, Billerica, MA). Following TUNEL staining, cells were immunostained with ZIKV-4G2 antibodies using the IFA protocol described above. The cells were visualized and imaged using a Keyence microscope (Keyence, Itasca, IL).

### Immunoblotting

Immunoblotting was performed using a method we described previously (63). Briefly, the treated and untreated cells were washed using ice-cold PBS and lysed using RIPA lysis buffer containing Halt protease and phosphatase inhibitor cocktail (Thermo Scientific, Rockford, IL). The total protein concentration was estimated using BCA protein estimation kit per the manufacturer’s instructions (Thermo Scientific, Rockford, IL). Forty micrograms of total protein were resolved on SDS-PAGE gels and transferred onto nitrocellulose or PVDF membranes. Following transfer, the membrane was blocked using a blocking buffer containing 5% non-fat skim milk in 1× TBST. Subsequently, blots were incubated with anti-rabbit/mouse primary antibodies (1:1,000 dilutions) diluted in 3% BSA prepared in 1× TBST, overnight at 4°C with gentle agitation. After incubation with the primary antibodies, the membranes were washed three times using 1× TBST, followed by incubation with anti-mouse or anti-rabbit HRP-conjugated secondary antibodies (1:2,000 dilutions) at RT for 1 h. Following three washes with 1× TBST, the blots were developed using Supersignal West Femto chemiluminescent substrate and imaged using iBright FL1500 imager (Thermo Fisher Scientific, Rockford, IL).

### RNA isolation and real-time qPCR

Following treatment, cells were collected in TRIzol reagent (#15596018), and the RNA was isolated per the manufacturer’s instructions (Thermo Scientific, Rockford, IL). RNA (1 µg) was reverse transcribed to cDNA using a Maxima first-strand cDNA synthesis kit, per the manufacturer’s instructions (Thermo Scientific, Rockford, IL). The cDNA was amplified using mouse or human gene-specific PCR primers in a 96-well plate using QuantStudio 3 Real-Time PCR system (ThermoFisher Scientific, Rockford, IL). The relative mRNA expressions of the gene of interest were normalized with respect to the housekeeping 18sRNA gene. The data were analyzed using the 2^−ΔΔ*C*_T_^ method and represented as relative fold change.

### Statistical analysis

The statistical analysis was performed using GraphPad Prism 10 V10.1.2 (GraphPad Software, La Jolla, CA). The one- or two-way ANOVA was used to compare the statistical differences between the experimental groups. A *P* value of <0.05 was considered statistically significant. The data were expressed as mean ± SD from three biological replicates unless indicated otherwise.

## Data Availability

All relevant data are within the article and figures.
